# Neural network assessment of herbal protection against chemotherapeutic-induced reproductive toxicity

**DOI:** 10.1186/1742-4682-9-1

**Published:** 2012-01-24

**Authors:** Amr Amin, Doaa Mahmoud-Ghoneim, Muhammed I Syam, Sayel Daoud

**Affiliations:** 1 Biology Department, UAE University, University St., Al-Ain 17551, UAE; 2 Physics Department, UAE University, University St., Al-Ain 17551, UAE; 3 Department of Mathematical Sciences, UAE University, University St., Al-Ain 17551, UAE; 4 Histopathology Laboratory, Tawaam Hospital in affiliation with Johns Hopkins medicine, Abu Dhabi Rd. Al-Ain 15258, UAE; 5 Zoology Department, Faculty of Science, Cairo University, Giza, Egypt; 6 Medical Physics Department, Tom Baker Cancer Center, Calgary, Alberta, Canada

## Abstract

The aim of this study is to assess the protective effects of *Ginkgo biloba*'s (GB) extract against chemotherapeutic-induced reproductive toxicity using a data mining tool, namely Neural Network Clustering (NNC) on two types of data: biochemical & fertility indicators and Texture Analysis (TA) parameters. GB extract (1 g/kg/day) was given orally to male albino rats for 26 days. This period began 21 days before a single cisplatin (CIS) intraperitoneal injection (10 mg/kg body weight). GB given orally significantly restored reproductive function. Tested extract also notably reduced the CIS-induced reproductive toxicity, as evidenced by restoring normal morphology of testes. In GB, the attenuation of CIS-induced damage was associated with less apoptotic cell death both in the testicular tissue and in the sperms. CIS-induced alterations of testicular lipid peroxidation were markedly improved by the examined plant extract. NNC has been used for classifying animal groups based on the quantified biochemical & fertility indicators and microscopic image texture parameters extracted by TA. NNC showed the separation of two clusters and the distribution of groups among them in a way that signifies the dose-dependent protective effect of GB. The present study introduces the neural network as a powerful tool to assess both biochemical and histopathological data. We also show here that herbal protection against CIS-induced reproductive toxicity utilizing classic methodologies is validated using neural network analysis.

## 1. Background

Cis-diamminedichloroplatinum (II) or cisplatin (CIS) is a highly effective anti-neoplastic DNA alkylating agent used to treat many types of solid tumors including testicular, ovarian, breast, lung, bladder, and head and neck. However, adverse side-effects, including testicular toxicity, limit its application [1,2]. Both short-term and long-term effects of CIS treatment on testicular function have been previously documented in human [2] and in other animal models [3,4]. Within days of CIS injection, animals develop severe testicular damage characterized by germ cell apoptosis, Leydig cell dysfunction and testicular steroidogenic disorder [4-7]. Germ cell apoptosis has been reported to play an important role in CIS-induced testicular damage [4-7]. CIS-induced DNA adduct formation in rat's spermatozoa was observed after treatment with CIS at a dose of 10 mg/kg body weight [8].

Free radicals have been reported to mediate reactions responsible for a wide range of CIS-induced side-effects. Consequently, anti-oxidants have been shown to protect non-malignant cells and organs against damage by CIS [8]. CIS has previously been shown to induce lipid peroxidation (LP) with a concomitant decrease in the level of testicular anti-oxidants [6].

*Ginkgo Biloba *(GB) has been used in traditional Chinese medicine for about 5000 years. It is one of the herbal drugs that has been widely used due to its antioxidant properties, ability to modify vasomotor function, effect on ion channels to inhibit activation of platelets and smooth muscle cells [9,10], stimulate neurotransmitters [11], decrease adhesion of blood cells to endothelium, and to modify signal transduction [9]. GB has also been used in the treatment of Alzheimer's disease and cognitive impairment. The major bioactive components of GB are flavonoglycosides and terpene lactones. GB extract was also reported for many decades to increase peripheral and cerebral blood flow as well as for the treatment of dementia. Furthermore, GB leaves extract have been shown to have strong antioxidant that directly scavenges reactive oxygen species (ROS) [11].

In the present investigation, GB is shown to attenuate the CIS-induced testicular toxicity in rats. CIS generates free radicals that react with key cellular components such as lipids, resulting in lipid peroxidation. Malondialdehyde (MDA) is one of the most known markers of lipid peroxidation that has widely been accepted as a measure of the cell injury [12]. Normally, the antioxidant defense system reduces the level of oxidative damage via a variety of enzymes such as catalase (CAT) and superoxide dismutase (SOD). GB extract (24% *Ginkgo biloba *flavonoglycoside, 6% Terpene lactones) has restored the normal level of both CAT and SOD and reduced the amount of lipid peroxidation and myeloperoxidase (MPO).

Inspired by the biological neural system, artificial neural network (ANN) has been introduced and used in medical and biological literature for many years; and has offered data mining solutions that have been proven quite useful among clinical researchers [13,14]. Neural networks have several unique characteristics and advantages as tools for a wide spectrum of applications. It has adaptive nature, where "learning by example" is replaced by "programming" in solving problems. This property renders such computational models viable in several applications, particularly where available information is scarce. The parallelization in the neural networks makes it easy to optimize the network to deal with a large volume of data and to analyze numerous input parameters.

In the last two decades, ANN has been universally utilized in medical application, science, and engineering. It works efficiently to use the prior information to respond to the new information rapidly and automatically. ANN is a good tool to employ and to deal with the time consuming and complex nonlinear relations especially when the traditional methods are difficult to be implemented. ANN has been recently gathering a great deal of interest [15] mainly due to its (i) Flexible use with non-linear modeling of large data set; thus, reducing the cost of the implementation and achieving higher processing speed and simpler implementation; (ii) Accuracy for productive inference which supports the clinical decision-making; (iii) Simplifying the dissemination of knowledge [16].

ANN has been developed as generalization of mathematical models of biological nervous systems. ANNs are mainly used in solving problems with extremely difficult or unknown analytical solution [17]. ANNs' property of learning from examples makes them powerful programming tools especially when domain rules are not completely certain or when certain amount of inaccuracy or conflicting data exist [18].

The effect of GB has been assessed using ANN implemented in classifier known as Neural Networks Clustering (NNC) using two types of data (i) Texture Analysis (TA) on the microscopic sections of testes and (ii) Biochemical & fertility indicators. TA has been proven to give diagnostic results in previous work done in our group [19,20]. Therefore, this method was used here for quantitative assessment of CIS-induced testicular toxicity. The biochemical indicators (such as MDA, CAT, SOD, and others) are the gold standard methods for evaluating the testicular damage/protection processes. A comparison between the NNC results of TA parameters and the biochemical indicators was carried out.

## 2. Materials and methods

### 2.1 Animals

Sixty male Wistar rats (120-240 g) were obtained from the Animal House, United Arab Emirates University. They were kept in polycarbonate cages and supplied with standard pellet diet and tap water under a 12 h light/dark cycle and room temperature of 22-24C. This study was approved by Animal Ethics Committee, UAE University.

### 2.2 Chemicals and Plant

CIS was a kind gift of Prof. Adeghate (FMHS, UAE University) and manufactured by Hospira UK Limited, Warwickshire, CV31 3RW, UK. GB leaves extract was purchased from General Nutrition Corporation (Pittsburgh, PA 15222, USA). Thiobarbituric acid, 1,1,3,3-Tetramethoxy-propan, phosphoric acid, sulfuric acid and hydrogen peroxide were obtained from Sigma Chemical (St. Louis, MO, USA).

### 2.3 Experimental Protocol

Rats were divided into six groups (n = 10). Groups were treated as followed: Normal (N); received saline (5 ml/kg body weight) and water (5 ml/kg body weight); CIS-induced (CIS); received a single dose (10 mg/kg, intraperitoneally); used previously to induce a clear testicular toxicity in various animal species [3,5,21,22]. GB was administrated orally (by gavages) alone at 200 mg/kg (GB); or for 21 days before CIS treatment at 200 mg/kg body weight (CIS + H); or at 100 mg/kg body weight (CIS + M); or at 50 mg/kg body weight (CIS + L). The three last groups were indicated in the text as CIS + GB-treated groups. GB was received one hour after CIS injection to avoid disturbance in absorption of each of CIS and GB. Five days after the administration of CIS, the rats were sacrificed after being anesthetized with diethyl ether. Rats were weighed regularly and their testes and epididymes were dissected out and weighed after sacrifice.

### 2.4 Biochemistry

Testes were homogenized separately in ice cold Tris-KCl buffer (150 mmol/L). Supernatants were collected and assayed for lipid peroxidation, CAT and SOD. Determination of MDA in testicular homogenate was based on its reaction with thiobarbituric acid (TBA) to form a pink complex with absorption maximum at 535 nm [23]. CAT activity was determined by measuring the exponential disappearance of H_2_O_2 _at 240 nm and expressed in units/mg of protein as described by Aebi [24] and total protein was estimated by the Lowry's method as modified by Peterson [25]. In testes, the SOD enzyme activity was determined according to the method described by Sun and Zigman [26]. This method was based on the ability of SOD to inhibit the auto-oxidation of epinephrine at alkaline pH to adrenochrome and other derivatives, which are easily monitored in the near-UV region of the absorption spectrum.

### 2.5 Histology

For histological examination, small pieces of testis were fixed in 10% neutral phosphate-buffered formalin and the hydrated 5 μm-thick sections were counter-stained with hematoxylin and eosin. Digital images were acquired under a Leica DMRB/E light microscope (Heerbrugg, Switzerland), at 40x magnification using an Olympus camera DP72. Images were acquired from 3 to 5 microscopic fields per animal which gave a total of 36 ± 4 image per group. Each field was digitized as a separate image of 744 × 744 pixels. The fields were selected such that boundaries and visible artifacts are avoided. Images were converted to bitmap (BMP) format, 24 bit, true color, RGB (Red, blue, and Green) pictures.

### 2.6 Texture Analysis and Neural Network classification

A neural network consists of a large number of simple processing elements called neurons. The network architecture is the arrangement of neurons into layers and the connection patterns within and between layers. Each neuron is connected to other neurons by means of directed communication links, each with an associated weight. The weights represent information being used by the net to solve a problem. Each neuron has an internal state, called its activation level, which is a function of the inputs it has received. An activation function is used to map any real input into a usually bounded range, often 0 to I or - I to 1 [27].

One can look at the ANNs as a function defined from domain to another domain or as a distribution over one or two domains associated with a particular learning algorithm. It is usually defined by the interconnection pattern between different layers of neurons, the learning process for updating the weights of the interconnections, and the activation function that converts a neuron's weighted input to its output activation. The nonlinear characteristic exhibited by neurons is represented by a transfer function.

**Figure 1:**
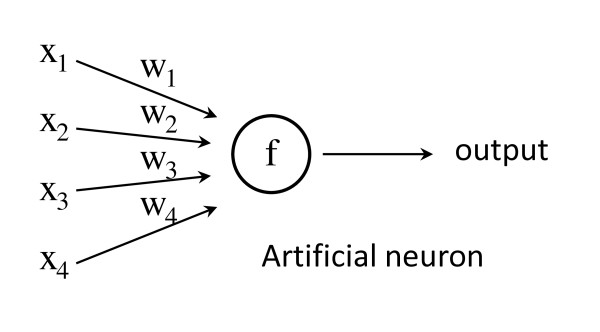
**Architecture of an artificial neuron**.

The idea of the artificial neuron can be described as follows. Let ***x***_**1**_**, *x***_**2**_,..., ***x***_***n ***_be ***n ***inputs that provide the neuron by unidirectional signal flows represented by the rows as in Figure 1 with weight vectors ***w*, *w***_**2**_, ..., ***w***_***n***_. Then, the activation transfer function is given by

$$output=f\left(net\right)=\left\{\begin{array}{cc} 1, & w^{T}x>\text{O̸}\\ 0, & w^{T}x\leq \text{O̸} \end{array}\right\}$$

where T is the transpose of the matrix, ∅ is called the threshold level,

$$\begin{array}{c} \;\;\;\;w=\left[\begin{array}{c} w_{1}\\ \vdots \\ w_{n} \end{array}\right],x=\left[\begin{array}{c} x_{1}\\ \vdots \\ x_{n} \end{array}\right],\text{ and}\\ net=w^{T}x=w_{1}x_{1}+w_{2}+x_{2}+\cdots +w_{n}x_{n}. \end{array}$$

Texture analysis as a non-destructive method of image analysis that provides hundreds of parameters which describe and characterize images based on special relationships between pixels. The combination of NNC with TA can provide a robust tool for image characterization and data mining; and therefore, can be utilized for evaluating the protective effect of GB.

Three methods of TA were applied on histological images belonging to N, CIS, CIS + L, CIS + M, and CIS + H groups, these are: Cooccurrence Matrix (COM), Run- length Matrix (RLM), and Gradient Matrix (GRM). These methods have been successfully applied on medical and microscopic images to discriminate between different experimental groups [18,28]. They are statistical in nature and depend on collecting quantitative information about image patterns that are inaccessible to the human eye. Details on the mathematical formula of these methods and calculation of their parameters can be found in literature [28].

NNC was applied to identify clusters based on two types of descriptors as mentioned above: Firstly, by biochemical [MDA, MPO, CAT and SOD] and fertility [Testis Weight (Testis W), Epididymis Weight (Epidid. W), Sperm Count (SpC), and Sperm Motility (SpM)] indicators; and secondly, by TA parameters. Due to the large number of TA parameters, an automatic selection method has to be applied in order to limit the number of descriptors to only those which have significance for characterization. For this purpose, the ten texture parameters of the highest *F-*coefficients were taken for further analysis. The *F*-coefficient is defined as the ratio of "between class variance" to "within class variance". TA parameters calculation and selection were performed using the software MaZda (version 4.5, ^©^1999-2006) [29].

The weighted average for each descriptor, whether biochemical or TA, was calculated based on the resulting clusters and shown graphically. Weighted average indicates the results of the difference between the mean value of one descriptor in its cluster (μ_d_) and its mean value in all clusters (μ_a_), divided by μ_a_.. NNC was performed using the software NeuroXL Clusterizer (OLSOFT LLC, ver 3.1.1, 2010).

### 2.7 Statistical analysis

Data are expressed as group mean ± SE. The statistical analysis was carried out using ANOVA, with SPSS version 10 (SPSS, Chicago, IL, USA). ANOVA was carried out to detect differences between all various groups. When significant differences were detected, analysis of a difference between the means of the treated and control groups was carried out using Dunnett's *t*-test.

## 3. Results

### 3.1 Histological effects of Ginkgo biloba

**Figure 2:**
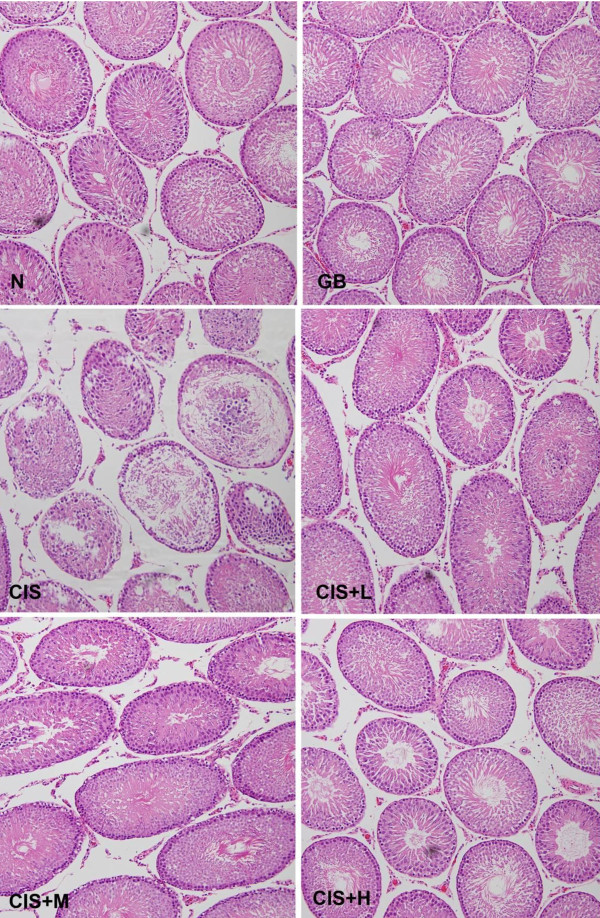
**Photomicrograph of cross sections of the testes of control (N), Cisplatin (CIS), *Ginkgo biloba *(GB) and CIS + GB-treated rats (Low dose of GB: CIS + L, Medium dose of GB; CIS + M and High dose of GB: CIS + H)**. Testes of N group show normal arrangement of germ cells and Sertoli cells. However, testes in CIS group show extensive atrophy of seminiferous tubules and degenerations of germ cells. Rats from CIS + L, CIS + M and CIS + H groups clearly show less degeneration of some tubules and irregular derangement of some germ cells (H & E 400x).

Control rats showed normal testicular architecture with an orderly arrangement of germinal and Sertoli cells. CIS treatment induced moderate to severe testicular atrophy with degeneration of germ cells in seminiferous tubules (Figure 2). The tubules were shrunken and greatly depleted of germ cells. There were depleted numbers of Leydig cells between the tubules. Sertoli cells with few germ cells were observed in the lumen. Animals pretreated with GB showed normal testicular morphology with irregular arrangement of germ cells and slight degeneration of seminiferous epithelium and shedding of germ cells in some tubules.

### 3.2 GB Effects on weights of testes and epididymis

The weights of testes and epididymes, expressed relative to the body weight, in rats after CIS administration were found to be significantly decreased, compared with the control group (Table 1). No significant changes in the weights of testes and epididymes were found in rats treated with GB before and after CIS treatment. The administration of CIS alone or along with tested herbs did not alter the body weight of the animals (data not shown).

**Table 1:** The effect of the *Ginkgo biloba *extracts on fertility-related parameters associated with CIS-treatment in testicular tissues of rats

Parameters	N	GB	CIS	CIS + L	CIS + M	CIS + H
Testis weight (gm)	3.51 ± 0.06	3.59 ± 0.11	2.99 ± 0.12*	3.49 ± 0.08•	3.52 ± 0.18•	3.65 ± 0.13•••

Epididymis weight (gm)	1.64 ± 0.06	1.53 ± 0.06	1.30 ± 0.04**	1.38 ± 0.05**	1.46 ± 0.08	1.44 ± 0.05

Sperm (count.10^6^)/gm of cauda	131.0 ± 7.73	134.9 ± 11.64	83.94 ± 8.03*	94.50 ± 20.25	94.67 ± 7.77	118.78 ± .8.78

Sperm Motility (%)	79.125 ± 3.34	68.12 ± 2.64	28.125 ± 1.52***	54.0 ± 5.59***•••	49.0 ± 3.83***•••	50.0 ± 4.2***•••

*P < 0.05 vs control, •P < 0.05 vs cisplatin, **P < 0.01 vs control, •••P < 0.001 vs cisplatin, ***P < 0.001 vs control

### 3.3 GB Effects on sperm motility and count

After CIS was administered, the epididymal sperm count and motility decrease significantly (P < 0.001) whereas sperm abnormality was increased (P < 0.001). Administration of GB attenuated the CIS-induced decrease of sperm count and motility and protected against sperm abnormality changes (Table 1).

### 3.4 GB Effects on testicular MDA and MPO

**Figure 3:**
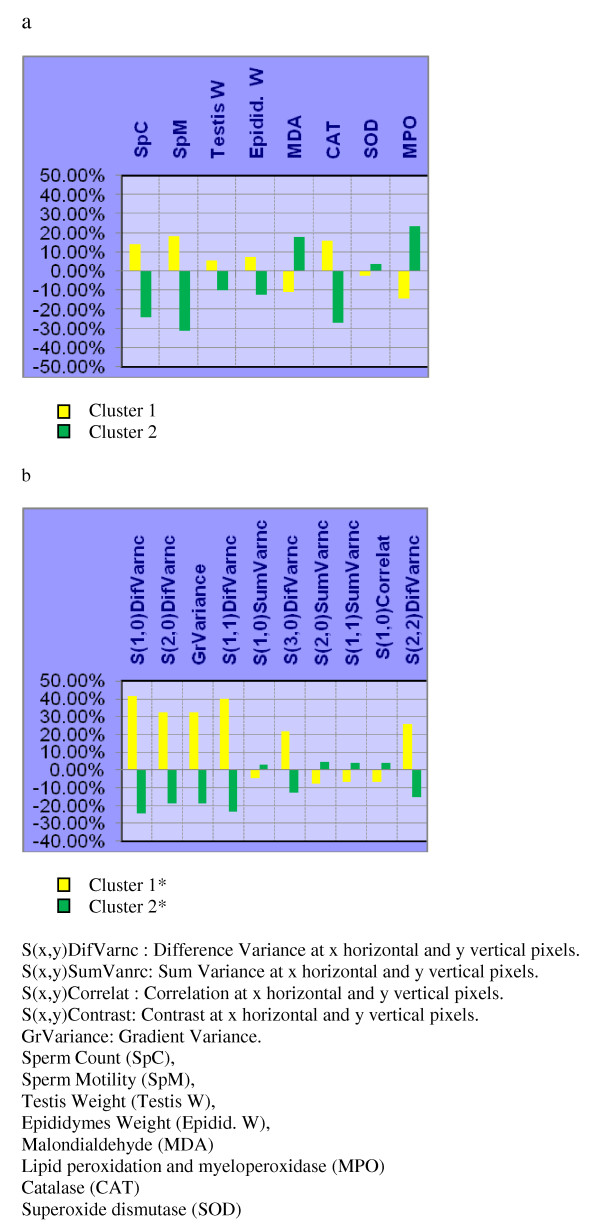
**Histogram representation of weighted averages of the individual descriptors of each cluster: a) Biochemical & fertility indicators; and, b) Texture Analysis parameters, on the groups: N, CIS, and CIS + H**. Each point represents the weighted average of either a biochemical & fertility or texture parameter descriptor.

A significant increase (P < 0.001) of testicular MDA was recorded after CIS treatment (Figure 3). Although MDA levels of pretreated animals (given GB before CIS treatment) did not return to the control level, there was no significant difference compared to the control.

GB treatment alone did not induce any change in the activity of MPO enzyme compared to the control group. In contrast, the CIS-treated group exhibited significant (p < 0.001) elevation in the MPO activity in the testicular tissue, compared to the control group (Figure 3). Significant effects on the MPO enzyme activity was also detected between the CIS + GB-treated groups and CIS-treated group.

### 3.5 GB Effects on testicular CAT and SOD

Testes of rats treated with CIS increased the SOD activity (p < 0.05). In contrast, the activity of CAT enzyme was significantly (p < 0.001) depleted in testicular tissues from this group of rats (Table 2). Interestingly, the treatment with GB restored normal activities of both SOD and CAT. Treatment with GB alone exhibited no significant effect on the enzyme activity of SOD and CAT compared with control group.

**Table 2:** The effect of the *Ginkgo biloba *extracts on oxidative stress parameters associated with CIS-treatment in testicular tissues of rats

Parameters	N	GB	CIS	CIS + L	CIS + M	CIS + H
MDA (nmol/mg protein)	0.97 ± 0.03	0.86 ± 0.08	1.2 ± 008***	1.0 ± 0.07c	0.84 ± 0.06a	0.98 ± 0.05c

MPO (mu/mg protein)	16.92 ± 0.34	17.13 ± 0.31	24.65 ± 2.2***	18.88 ± 0.93b	19.69 ± 1.54c	16.93 ± 0.59c

CAT (u/mg protein)	146.79 ± 2.9	144.07 ± 2.03	84.89 ± 3.24***	127.9 ± 3.9***a	118.0 ± 3.64***a	134.4 ± 1.88*a

SOD (u/mg protein)	3.37 ± 0.02	3.37 ± 0.01	3.62 ± 0.05***	3.26 ± 0.08a	3.16 ± 0.09a	3.25 ± .0.06a

Values are expressed as mean ± SEM of eight rats per group. Concentration is expressed as nmol/mg protein for MDA. Activity is expressed as unit/mg protein for CAT and SOD. Activity is expressed as m unit/mg protein for MPO. Significance was determined by one-way analysis of variance followed by Dennett's t test:* P < 0.05; ****P < 0.001 vs control. ^c^P < 0.05; ^b^P < 0.01; ^a^P < 0.001 vs. CIS group.

### 3.6 Neural Network Clustering of biomedical and fertility indicators and texture analysis parameters

The characteristic TA parameters that were selected using *F*-coefficient included 9 parameters from the COM and only one from GRM. None of the RLM parameters was represented in this selection. NNC was applied independently on the biochemical and fertility indicators or TA parameters.

**Figure 4:**
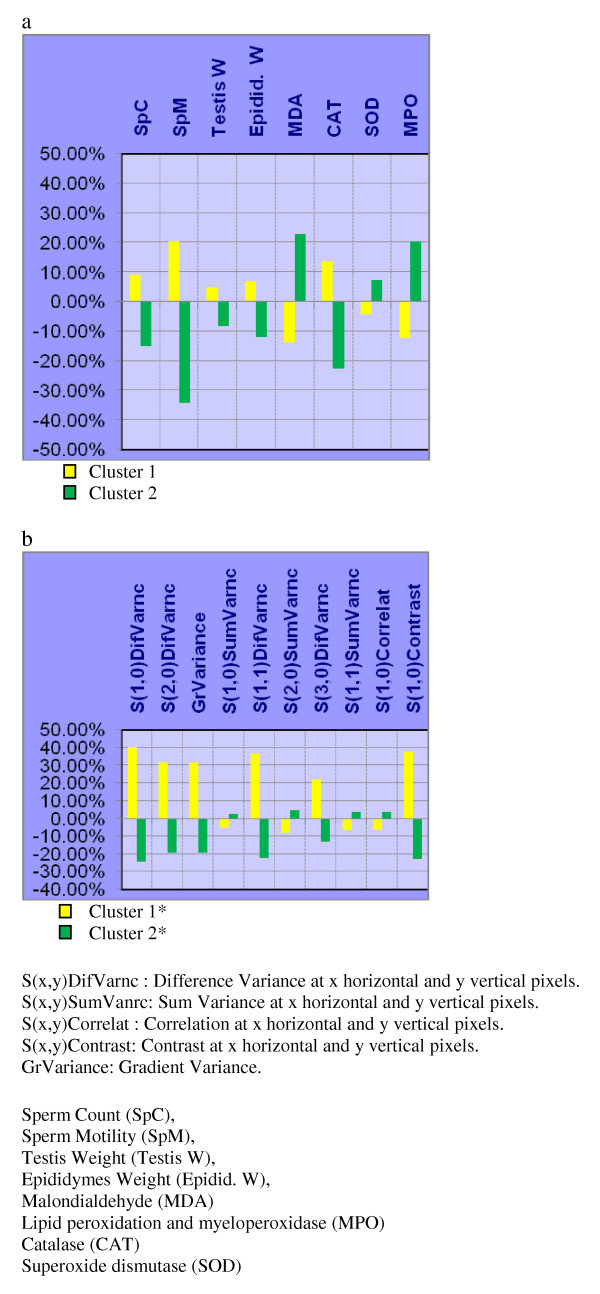
**Histogram representation of weighted averages of the individual descriptors of each cluster: a) Biochemical & fertility indicators; and, b) Texture Analysis parameters, on the groups: N, CIS, and CIS + M**. Each point represents the weighted average of either a biochemical & fertility or texture parameter descriptor.

**Figure 5:**
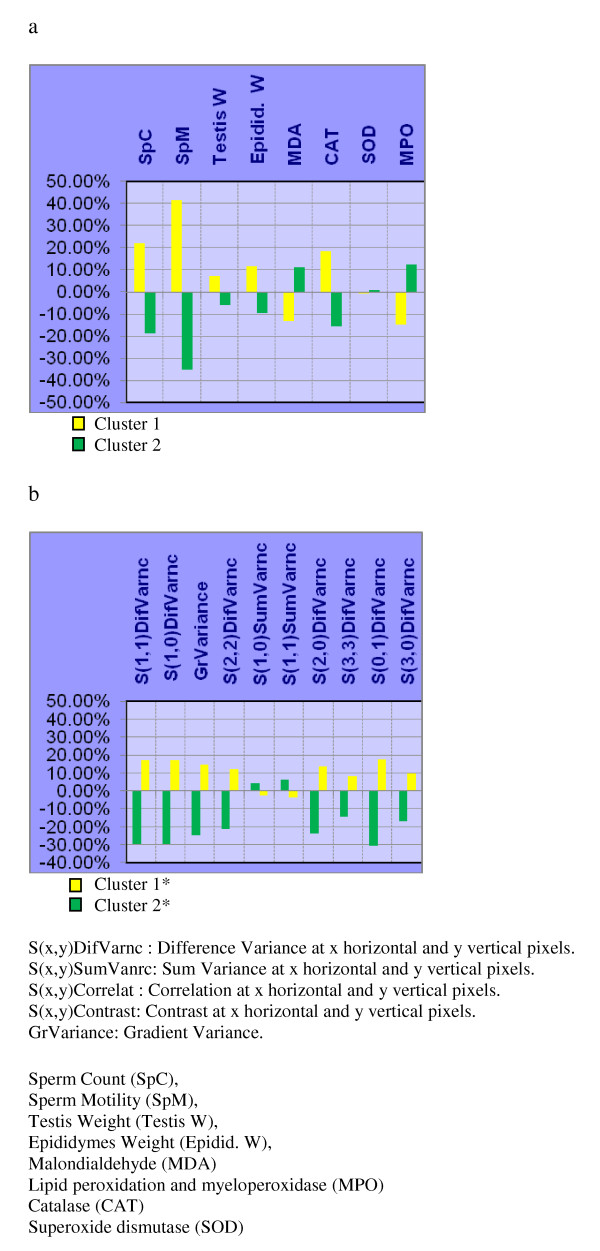
**Histogram representation of weighted averages of the individual descriptors of each cluster: a) Biochemical & fertility indicators; and, b) Texture Analysis parameters, on the groups: N, CIS, and CIS + L**. Each point represents the weighted average of either a biochemical & fertility or texture parameter descriptor.

In each NNC attempt, one of the groups: CIS + H, CIS + M, or CIS + L was tested for clustering against N and CIS which were considered to be the two reference groups dominating either clusters. The distribution of samples among the two clusters is shown in Table 3. Weighted averages of the individual descriptors belonging to biochemical and fertility indicators and image TA parameters are also shown in figures 3, 4, and 5.

**Table 3:** NNC results showing sample distribution among clusters

	Biochemical & fertility indicators		TA parameters	
	
	Cluster 1%	Cluster 2%	Cluster 1*%	Cluster 2*%
**N**	100	0	14	86
**CIS**	0	100	95	5
**CIS + H**	87	13	0	100
**CIS + M**	87	13	0	100
**CIS + L**	37	63	81	19

The values represent the percentage of samples from N, CIS, CIS + H, CIS + M, or CIS + L groups that were classified in either cluster 1 or 2 (or 1* and 2*). The cluster is assumed to represent one of the two main trends (normal: N, or pathologic: CIS) depending on the dominance of the group (N or CIS) in that cluster. For example Cluster 1 in biochemical & fertility Indicators is considered to be the reference normal cluster as it is dominated by N group. For TA parameters, Cluster 2* is considered to be the normal reference cluster for the same reason.

TA: Texture Analysis

As can be shown from NNC on biochemical & fertility indicators (Table 3), 100% of the group N samples aggregate in cluster 1 while 100% of CIS samples aggregate in cluster 2. However, groups CIS + H, CIS + M, and CIS + L showed differential distribution between the two clusters: 87% of CIS+H samples aggregated with N in cluster 1 (Table 3). Similarly87% of CIS + M samples aggregated in cluster 1 (Table 3), while the remaining 13% from each group aggregated with CIS in cluster 2. On the other hand, only 63% of CIS + L samples aggregated in cluster 1 and 37% in cluster 2 (Table 3). The NNC using TA parameters also showed that N, and either CIS + H or CIS + M, tend to aggregate together in cluster 2* (Table 3); while CIS + L aggregated mainly with CIS in cluster 1* (Table 3).

## 4. Discussion

NNC showed that CIS + H group aggregates with N group in one cluster which indicates that both groups have similar biochemical properties and image texture parameters which are different from CIS group. This demonstrates that GB at high dose is effective in protecting the testes from degenerative effects induced by CIS. Medium dose of GB can be regarded in the same way, as the CIS+M group also clustered mainly with N group. On the other hand, low dose of GB shows less aggregation with N group and more aggregation with CIS group; and hence, less effective protective effect.

The result of NNC on TA parameters has particularly interesting interpretation as it showed aggregation of GB treated samples with N cluster (cluster 2*) except for CIS + L which mainly aggregated with the CIS group in cluster 1* (Table 3). This can be explained by referring to the way TA works on images: For TA, an image is a group of quantitative patterns and statistical relationships; the presence of common patterns or relationships in an experimental group is seen by TA as being a unique texture that characterizes this group from other textures or probably matches this group to similar textures. The images of CIS + L group demonstrate visible and salient degenerative patterns that make this group's texture similar to CIS and different from N (Figure 2); and hence, the clear cut classification of CIS+L with the CIS dominant cluster (cluster 1*) (Table 3).

NNC is an unsupervised classification method applied without giving the classifier information about the sample's original group, which makes clustering output reliable. In addition, as an unsupervised method, NNC does not require testing; and therefore, it is faster and can be used for classification when the number of samples is limited. The consistency of classification results between TA parameters and biochemical & fertility indicators suggest that the protective effect of GB does not only appear as biochemical effects but also as histological patterns detectable by TA. In all cases the protective effect of GB against CIS-induced testicular toxicity is dependent on the GB dose used.

Normally, administration of CIS results in overproduction of free radicals and thus causing an oxidative stress which eventually leads to reproductive toxicity. Oxidative stress contributes to big extent to testicular damage and is generally associated with spermatogenic damage and germ cell apoptosis. It has been observed that GB eliminated oxidative stress by increasing antioxidant potential [30-33]. To our knowledge, this is the first study to provide evidence that administration of GB decrease the cytotoxic damage imparted by CIS drug in rat's reproductive system. Recent studies have shown that herbal plant extracts protect against CIS-induced reproductive damages due to the presence antioxidant agents in those plants [34,35]. While CIS is one of the leading anticancer drugs in the chemotherapy treatment of variety of cancer types, it induces a testicular damage, sperm dysfunction, germ cell apoptosis and abnormalities in Lyedig cells in rats. As lower mg/kg doses of CIS are routinely used clinically, lower doses will be investigated to assess GB prophylactic or therapeutic value.

This report illustrates a significant decrease in the level of CAT enzyme compared to control. CAT has been shown to reduce the free radicals and ROS produced by normal cells metabolites. It has also been shown that CIS generates free radicals which interfere with the antioxidant defense system. Therefore over production of ROS, which normally leads to lipid peroxidation, results in increase in MDA levels as shown in this study.

Recently, there have been increasing numbers of publications that reveal the antioxidant functions of many plant extracts. However, a little is known about protective agents against CIS-induced testicular damage. The present investigation illustrates that the administration of GB extract, restores the control values of oxidative stress markers. This study provides evidence that the antioxidative properties of GB may contribute to its ability to restore the level of CAT enzyme and to reduce the MDA content. The antioxidant activity of GB could be attributed to its active components namely, flavonglycoside and terpene lactones. Further investigations are underway to unravel the molecular mechanism underlying the GB-protective effect against CIS-induced reproductive toxicity. We will also soon examine whether or not the GB prophylactic effect could be enhanced if applied in combination with other well-known antioxidants such as vitamin C and/or E.

## Competing interests

The authors declare that they have no competing interests.

## Authors' contributions

AA designed the in vivo study, supervised the collection of biochemical and fertility data, contributed to the writing and the revising of the manuscript. DMG designed and executed the neural network analyses, contributed heavily to the writing and the revising of the manuscript. MS contributed to the statistical analysis and to the processing of the manuscript for publication. SD carried out the histological analysis. All authors read and approved the final manuscript.
